# Outbreak Investigation of Typhoid Fever in the District of Gabes, South of Tunisia

**DOI:** 10.3390/epidemiologia4030023

**Published:** 2023-06-23

**Authors:** Aicha Hechaichi, Hind Bouguerra, Hajer Letaief, Mouna Safer, Lamia Missaoui, Amal Cherif, Saffar Farah, Houcine Jabrane, Taoufik Atawa, Hamdi Yahia, Hayet Hamdouni, Khadija Zitoun, Karim Chahed, Ramzi Laamouri, Jaber Daaboub, Mohamed Rabhi, Afif Ben Salah, Mohamed Kouni Chahed, Nissaf Bouafif Ben Alaya

**Affiliations:** 1 National Observatory of Emerging Diseases, Ministry of Health, Diplomat Building, 5-7 Khartoum Street, Le Belvédère, Tunis 1002, Tunisiahejerletaief@gmail.com (H.L.); safermouna@gmail.com (M.S.); missaouilamia88@gmail.com (L.M.); amalcherif206@gmail.com (A.C.); farah.saffar@outlook.fr (S.F.); dr.karimchahed@gmail.com (K.C.); ramzi.laamouri@gmail.com (R.L.); 2 Preventive Medicine Department, Faculté de Médecine de Tunis, Université de Tunis El Manar, Tunis 1007, Tunisia; mchahe@yahoo.fr; 3 Preventive Medicine Department, Faculté de Médecine de Tunis, Université de Tunis El Manar, LR01ES04 Epidémiologie et Prévention des Maladies Cardiovasculaires en Tunisie, Tunis 1007, Tunisia; 4 Direction Régionale de Santé Gabès, Place du Gouvernorat, Gabès 6000, Tunisia; houcinejabrane2016@gmail.com (H.J.); yahia.hamdi@yahoo.fr (H.Y.); 5 Direction des Soins de Santé de Base, Ministry of Health, 31 Khartoum Street, Tunis 1002, Tunisia; atawataoufik78@gmail.com (T.A.); hamdounihayet@gmail.com (H.H.); 6 Direction Régionale de Santé Tunis, 9 Rue Ibn El Haythem, Tunis 1002, Tunisia; khadija.zitoun97@gmail.com; 7 Direction de L’hygiène du Milieu et de la Protection de L’environnement, Ministry of Health, Bab Saadoun, Tunis 1006, Tunisia; jadaaboub@yahoo.fr (J.D.); mohamed.rabhi@rns.tn (M.R.); 8 Graduate Studies and Research, Arabian Gulf University, Road 2904 Building 293, Manama 329, Bahrain; afifbs@agu.edu.bh

**Keywords:** typhoid fever, disease outbreak, epidemiology, risk factors, waterborne infection

## Abstract

Typhoid fever is a significant public health concern in many parts of the world, particularly in developing countries with poor sanitation and hygiene conditions. In July 2016, an outbreak of typhoid fever occurred in Ghannouche, located in the south of Tunisia. This paper reports the results of a field investigation undertaken to identify possible transmission pathways and risk factors in order to propose control and preventive measures. A retrospective cohort study including a passive and active case finding, as well as an environmental and bacteriological investigation was conducted from July to September 2016. A case was defined as a person residing or having stayed in Ghannouche and having presented from the beginning of June clinical signs suggestive of typhoid fever, with, for a confirmed case, laboratory isolation of S.Tyhi, and for a probable case, an epidemiological link with a confirmed case. Attack rates were determined, and risk ratios were estimated with respect to exposures. Unadjusted and adjusted odds ratios were estimated using binary logistic regression. Among the 628 subjects investigated, 102 cases of typhoid fever were identified (74 confirmed and 28 probable) with an overall attack rate of 16.24%. Over 56% of cases were male and those under 10 years old were most affected (38.2%% of cases) with a median age of 12 years (interquartile range 5 to 25 years). The main clinical signs were fever (95%) and diarrhea (57%). Young age (adjusted OR = 0.95 and 95% CI = 0.93–0.97), low level of education (adjusted OR = 4.76 and 95% CI = 1.34–16.81), and the habitat type Arab or rudimentary house (adjusted OR = 4.93 and 95% CI = 2.61–8.27) were the socio-demographic factors independently associated with typhoid fever. Typhoid fever was found to be associated with drinking softened water (adjusted OR = 2.64 and 95% CI = 1.16–4.82), eating raw fruit and vegetables from family gardens (adjusted OR = 6.13 and 95% CI = 3.66–11.06), and using uncontrolled waste disposal (adjusted OR = 3.52 and 95% CI = 2.03–6.94). A total of 110 drinking water samples were analyzed; out of the 38 samples of softened water, 12 were non-compliant and 5 were positive for *Salmonella*. The screening activity identified two asymptomatic carriers, one of whom was a softened water seller. We concluded that drinking softened water from informal or unauthorized sale units, consuming fruit and vegetables from family gardens, uncontrolled dumping of household waste, and poor socio-economic conditions increase the risk of typhoid fever in this region. Many recommendations were implemented to stop this outbreak and to prevent further episodes.

## 1. Introduction

Typhoid fever is a systemic bacterial infection caused by *Salmonella enterica* serotype typhi (S. typhi) that begins in the digestive tract and then spreads throughout the body. The natural reservoir and host of the bacteria is exclusively the human body. Transmission results from the ingestion of water or food contaminated by the feces of infected people or from human-to-human transmission [1]. The incubation period ranges from three days to two months [2]. The illness presents with prolonged fever associated with other symptoms including pulse-temperature dissociation, fatigue, anorexia, headache, abdominal pain, and diarrhea or constipation [3]. Without therapy, the illness may last for 3 to 4 weeks and death rates range between 10% and 30%, but decline to 1–4% with an appropriate and timely antimicrobial treatment [4]. Notably, a small number of individuals, known as chronic carriers, can carry and release bacilli in their stool or urine for more than 1 year either continuously or intermittently, and chronic carriage is noted in 2–5% of cases for S. Typhi [2].

According to WHO estimates, 21.6 to 26.9 million cases of typhoid fever with more than 216,000 deaths occur each year worldwide [4]. Typhoid fever is prevalent in most countries, but remains endemic in developing countries with low levels of hygiene, constituting a significant public health problem [4,5].

In Tunisia, typhoid fever is a national notifiable disease with a low national incidence of 0.32 cases per 100,000 population in the last five years, and an average of 35 sporadic cases reported per year. It is still endemic–epidemic, especially in rural areas, where drinking water and sanitation are lacking. Its surveillance is based on the double declaration of any case of typhoid fever from the peripheral to the regional level, then to the central level with the immediate reporting and investigation of any grouped cases and epidemic events of typhoid fever.

Over a dozen confirmed cases of typhoid fever were reported in a small city in the south of Tunisia named Ghannouche, our study location in July 2016. Given this high number, exceeding the average number of cases reported annually across the country, the epidemic alert was given. Following this alert, a crisis unit was established at regional and central levels and a joint rapid response team was mobilized to conduct the field investigation to identify the potential source of the outbreak and to implement effective control and preventive measures.

The current study presents the results of the epidemiologic and environmental investigation conducted to identify the determinants and the risk factors associated with this outbreak in order to inform timely and effective control measures.

## 2. Methods

### 2.1. Epidemiological Investigation

The study was carried out from 1 August to 6 September 2016, and it was undertaken in three main phases: (1) Active search for all cases of typhoid fever diagnosed in the governorate of Gabes during the investigation period and any other case that may have an epidemiological link with the current episode to identify the index case. (2) The description of the epidemic episode by conducting a descriptive survey integrating the data of the active search for cases and the passive notification of cases through the Notifiable Diseases Reporting System. (3) The analysis of the epidemic episode by conducting a retrospective cohort study to identify the risk factors and determinants of this epidemic episode.

Through contact tracing, we were able to draw the limits of the affected area from which cases came: The area in question was limited and included three major cities that are part of the Ghannouche delegation. This region is an area known for its precarious environmental situation given its proximity to a chemical complex, several housing complexes not being connected to public drinking water and sanitation as well as the concept of softened water consumption from water desalination units, which are clandestinely installed in the region.

For an effective case finding, we chose at the beginning a case definition with three levels of sensitivity and specificity (suspected case, probable case, and confirmed case). We defined a suspected case as a resident or visitor to the delegation of Ghannouche, with onset on or after the beginning of June 2016, who presented at least one of the following signs and symptoms: torpor, diarrhea, constipation, vomiting, and abdominal pain. A confirmed case required the biological isolation of S. Typhi from blood, bone marrow, bowel fluid, or stool. A probable case was any suspected case with an epidemiological link to a confirmed case. At the end of the investigation, we grouped those who met the confirmed or probable case definition as people with typhoid fever, and we considered the suspected cases as healthy subjects after negative laboratory results and the subjects carrying S Typhi without clinical signs as asymptomatic carriers.

Cases were retrospectively recruited from the data of the Notifiable Diseases Reporting System, and prospectively by an active search for symptomatic cases in the pediatric or infectious diseases wards, in coordination with the bacteriology laboratory of the regional hospital, or in health centers. In addition, a face-to-face investigation was implemented in the immediate family circle and/or community of identified confirmed cases to identify symptomatic cases not reported by the passive system and to detect healthy carriers.

We included in our study all households in the affected district regardless of age, gender, geographic origin, and length of stay. A standardized questionnaire on socio-demographic factors and clinical characteristics, location information (GPS coordinates), time information (date of onset of signs, date of diagnosis, and hospitalization), clinical progression, and possible sources of contamination during the month preceding the onset of symptoms was orally administered by trained staff from the primary health centers and hospital hygiene department.

Data analysis was performed using SPSS 18; it includes a descriptive study with a description of the whole study population and a description of the outbreak in terms of time, place, and individual case characteristics by calculating the proportion of cases and the specific attack rates. We have described the outbreak in terms of place according to the GPS coordinates of cases (place of residence) that were collected and analyzed using Geographic Information System ([“ArcGIS”]) to study the geospatial evolution of cases and the potential spread of the outbreak to other regions.

In addition, an analytical study was conducted to study the relationship between the occurrence of typhoid fever, which is the event of interest, and the exposure factors collected during the survey, concerning mainly sources of drinking water, water management, sanitation system, and waste management and also sources of fruit and vegetables.

For statistical analysis, univariate analysis was carried out first using the Pearson chi-square test to compare the qualitative variables and Student’s *t*-test to compare the quantitative variables. The association measures were made by calculating the crude odds ratio (OR) and its 95% confidence interval using the simple binary logistic regression method. Then, we conducted a multivariable analysis applying the binary logistic regression technique following the step-by-step descending method. Only variables with a *p*-value of <0.20 in univariate analysis were introduced in the multivariable analysis. The interaction between the different variables was tested in bivariate analysis based on the homogeneity test. In the final model, we selected variables independently associated with the variable of interest with a *p*-value of ≤0.05. Adjusted OR (AOR) presented with their 95% confidence intervals measured the strength of the association between exposure factors and the event of interest.

### 2.2. Bacteriological and Environmental Investigation

Drinking water, food, sewage, and stool specimens were collected from home cases and the surrounding area in the search for pathogens, particularly salmonella. An inspection of the catering staff, food, and water outlets in the study area was also carried out by the local hygiene unit with samples of food, water, and stool, looking for contaminated food and asymptomatic carriers among staff.

The clinical strains of S. Typhi have been isolated at the regional microbiology laboratory. Characterization of isolated strains, comparison between them, and serotyping were performed at the national microbiology laboratory. Samples of water and food ingredients were analyzed at the regional laboratory of hygiene and the national laboratory of salmonellosis at the Pasteur Institute of Tunis, in order to compare the clinical strains (human) and those isolated in the environment.

### 2.3. Ethical Considerations

We have ensured the confidentiality, anonymity, and privacy of personal data, and the right to refuse participation. Oral informed consent was obtained before carrying out investigations with people. We did not use written consent because there was a risk that we would not respond quickly enough to such an emergency situation, and going through the standard ethics review process can take months to complete. In addition, the health and hygiene information of the local population and the therapeutic care of screened subjects were guaranteed.

## 3. Results

### 3.1. Descriptive Epidemiology

#### 3.1.1. Description of the Study Population

During the study period, we investigated 628 subjects distributed across 170 households. These households share common environmental characteristics; 62 of them (36.5%) were not supplied with tap water coming from the central laboratory of the National Water Supply and Distribution Company but were supplied by other water sources such as wells, tanks, natural sources, or softened water. Concerning water consumption practices, 67.7% of respondents were using softened water for drinking while national tap water was mainly being used by only 19.7%. The assessment of bacteriological water quality by the health authorities has not been realized in 88.2% of cases. Free residual chlorine in drinking water has been measured for only 135 households, which represents 79.4%; this measure showed that the absence of chlorine was observed in 35 households, about 26% of cases.

Regarding wastewater sanitation and waste management, 51.9% of respondents reported that their habitat was connected to the public sanitation system and 45.2% used cesspools, among which 19.7% were overwhelmed. The evacuation of household waste by the municipal departments was the most current method in 84.6% of cases. Our study revealed that 96% of the respondents consumed fruit and vegetables bought from the local market and 20.4% consumed those from the family gardens, among whom 11% are irrigated by wastewater.

#### 3.1.2. Description of the Outbreak

**Figure 1:**
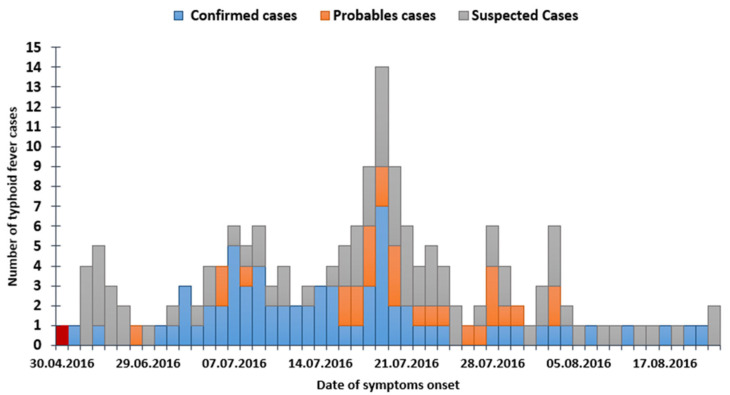
Epidemic curve of typhoid fever cases according to their status and date of symptoms onset, Ghannouche Gabes on 6 September 2016.

Of the total 628 persons investigated, 187 persons meet the first case definition criteria; among these, 74 were confirmed, 28 probable, and 85 suspected cases, and the remaining were healthy subjects. The first case of a patient diagnosed with typhoid fever detected during this epidemic was reported on 10 June 2016 (week 24) (Figure 1), an index case confirmed by laboratory diagnosis. The following emerging cases occurred between 20 and 30 June with an epidemiological link to the index case. The outbreak appeared to peak on 19 July 2016 (week 30). The number of cases afterward gradually declined with the implementation of the control and preventive measures from early August 2016. The last confirmed case was reported on 1 September 2016 (week 36).

**Figure 2:**
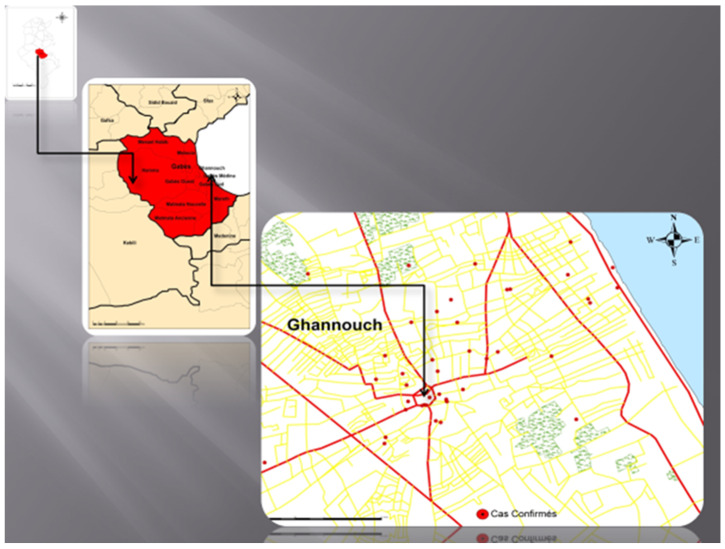
Distribution of confirmed cases of typhoid fever, Ghannouche July–September 2016.

We only traced and recorded the GPS coordinates of the residential addresses of confirmed case patients recorded in the line list: 68 (92%) in Ghannouche, representing the most important cluster. The remaining cases belonged to other delegations of the governorate of Gabes (Hamma, Gabes City, Mareth) (Figure 2).

According to the most specific case definition, chosen later, 102 were retained as typhoid fever cases (74 confirmed and 28 probable) and we considered all the suspected cases as healthy subjects after negative laboratory results. The overall attack rate of typhoid fever was 16.24% (95% CI [13.2–19.3%]). The median age of the 102 case patients was 12 years (interquartile range: 5–25 years). The most affected age group was less than 10 years old in 38.2% of cases. There was a trend of decreasing age-specific attack rates with increasing age. Males recorded a higher attack rate than females (Table 1). Most cases recorded had a low socioeconomic status: they had a primary level of education in 40.2% of cases and lived in Arab or rudimentary houses in 78.4% of cases. The clinical profile consisted of fever, the most constant sign (95%), and digestive signs such as diarrhea (57%) and abdominal pain (47%). The other classic signs of the disease, such as lenticular rosy spots and tuphos, were rarely revealed (Table 2). The hospitalization rate was 72.5% of cases. The majority of patients were treated with a third-generation cephalosporin (67.8%) or fluoroquinolones (30%), with an average treatment time of 10 days, and recovery for 96% of cases. Only two complications were reported: hepatitis and purulent meningitis among two children aged 3 years and 14 years, respectively. No deaths occurred during this epidemic.

### 3.2. Analytical Epidemiology

#### 3.2.1. Main Risk Factors for Typhoid Fever

Univariate analysis (Table 3) showed that the main socio-demographic factors associated with the disease were the age groups, especially the under-10 age group; the level of education, namely children out of school or subjects with a primary education level; and the type of housing, namely the Arab or rudimentary house. As regards environmental factors (Table 3), we noted that the drinking water source was significantly associated with the disease (*p* = 0.002) and the risk of disease was significantly higher (*p* = 0.001) among people who consumed softened water. In addition, poor storage and water conservation conditions are significantly associated with the occurrence of typhoid fever. For wastewater treatment, non-connection to the public sanitation system was significantly associated with the disease (*p* = 0.001) and people who used cesspools were more exposed to the disease compared to those who did not use this type of wastewater disposal. Notably, uncontrolled waste disposal was significantly associated with the disease. Some eating habits were significantly associated with the disease, such as the consumption of fruit and vegetables from the family garden.

Multivariate analysis (Table 4) identified the independent factors associated with typhoid fever. These factors were classified into two categories: socio-demographic factors and environmental factors associated with typhoid fever. Among the socio-demographic factors, age was a protective factor against typhoid fever; younger persons were at greater risk of becoming ill with this disease (adjusted OR = 0.95 and 95% CI = 0.93–0.97) and a low level of education was a risk factor, with the most associated category being never-enrolled people, with adjusted OR = 4.76 and 95% CI = 1.34–16.81. The habitat type Arab or rudimentary house was also significantly associated with the disease, the corresponding adjusted OR being 4.93 (95% CI [2.61–8.27]); the risk of being infected with salmonella was multiplied by 4.93 among persons who lived in Arab or rudimentary houses. Among environmental factors, drinking softened water was a risk factor for enteric fever; the risk of developing typhoid fever was multiplied by 2.64 (95% CI [1.16 to 4.82], *p* = 0.018) among persons who drank softened water compared to those who did not use this source of water supply. It has also been found that the consumption of fruit and vegetables from family gardens was also a risk factor for the disease, and persons who consumed fruit and vegetables from family gardens were more likely to catch the disease (adjusted OR = 6.13 and 95% CI = 3.66–11.06). Finally, it was noted that using uncontrolled waste disposal was independently associated with typhoid fever, with adjusted OR = 3.52 and 95% CI = 2.03–6.94.

#### 3.2.2. Laboratory Investigation

Analyses conducted on human strains have revealed the presence of *Salmonella enterica* serotype Typhi (OMA+, 0.9+, H: d+) with a wild phenotype (sensitive to antibiotics).

We performed 421 systematic stool cultures for family members of cases, two of whom were positive for salmonella; these were two healthy carriers, and one of whom was a softened water seller. We collected 110 water samples taken from different sources of drinking water and 59 samples of foodstuffs, including salads, vegetables, fruit, poultry, and raw milk. Of the 38 samplings of softened water, 12 were non-compliant with Tunisian standards in force (31.57%) and 5 were positive for salmonella.

## 4. Discussion

This investigation documented an extended outbreak of typhoid fever that occurred in Ghannouche, a small city in the south of Tunisia. The affected area had a particular environmental characteristic related to insufficient water supply and poor sanitation. In fact, 36.5% of households did not have public tap water and were supplied by open and uncontrolled water sources. Among household members of the affected area, 67.7% used softened water for drinking, while public tap water was used by only 19.7% of people. This trend to not use tap water even if it were present would be related to the discomfort with the taste due to the quality of this water and its sometimes unusual aspect and color. The discomfort with the taste of tap water is mainly related to its hardness because of its calcium and magnesium content, and the color change is mainly due to disturbances that occur in the water supply network such as leaks, repairs, or because of the effects of pipe aging. For all these reasons, many people consume softened water for drinking. Water softening is the process of removing the dissolved calcium and magnesium salts that cause hardness in tap water and replacing them with sodium ions [6]. As regards sanitation, only 52% of surveyed people reported that their habitat was connected to the national sanitation network; the others used wells, septic tanks, or open spaces. We also found the practice of fruit and vegetable consumption from family gardens irrigated by wastewater. The analysis of environmental conditions of the exposure population suggested the hypothesis of a waterborne epidemic.

The epidemic curve showed an irregular profile: cases or grouped cases occurred at variable intervals over a relatively long period of about 3 months, attesting repeated exposures, with rapid ascent and slow descent. The shape of the epidemic curve was suggestive of an intermittent common source poorly controlled followed by a person-to-person transmission [7,8]. The probable exposure period has been estimated from June 7th to July 1st. The decrease in the number of cases during the first week of August would be explained by the intervention of the field team followed by the implementation of control measures.

During this outbreak, the overall attack rate was 16.24%. It was higher than those observed in outbreaks reported in other studies conducted elsewhere [9,10,11,12], and it was higher among men than women (18.3% versus 14.1%). However, a female predominance has more frequently been reported in the literature [9,10,11], suggesting an increased risk of exposure through household activities, including the preparation of potentially contaminated raw foods and the care of children who are more likely to catch an infection. We also observed that there was a trend of decreasing age-specific attack rates with increasing age; these rates were high among younger age groups. This trend is typical in typhoid-endemic areas [13].

In this study, independent risk factors for typhoid fever were mostly related to certain socio-demographic factors and environmental conditions. Among socio-demographic factors, we found a significant negative association between age and the disease that could be explained by the immunization of older people either naturally, by acquiring an infection during childhood, or with anti-typhoid vaccination included in the Tunisian military vaccination schedule. These findings were observed in a case-control study conducted in Jakarta, Indonesia by Albert M. Vollard et al. [14], where age was found as a protective factor against typhoid fever, with adjusted OR = 0.96 and 95% CI = 0.94–0.98. A low level of education was also independently associated with typhoid fever, a concept supported by another study conducted by H. Tran et al. [15], where no education was independently associated with typhoid fever, with adjusted OR= 2.0 (95% CI [1.0–3.7], *p* = 0.03). In fact, through education, we acquire good hygiene practices and prevention methods. We also found that living in an Arab or rudimentary house was significantly associated with typhoid fever. This association could be explained by the poor living conditions associated with this type of habitat, such as inadequate hygienic habits and crowded households, that have been amply shown in the literature [14,16,17].

In this study, exposure to environmental factors was also significantly associated with typhoid fever. Drinking softened water was associated with an increased risk for typhoid fever in multivariate analysis, suggesting that this is an independent risk factor for this disease. Unsafe softened water, inadequately abstracted and stored using equipment in poor conditions was sold without control in a number of clandestine units all over the city. This indicator was easy to interpret, as explained in other investigated outbreaks of typhoid fever in Zimbabwe by Muti et al. [18] and in Uganda by Kabwama et al. [19], where uncontrolled water from unprotected sources sold on the street in small plastic bags was an independent factor associated with enteric fever. In light of this finding, it is important to consider the relationship between our study and other relevant studies conducted in developing countries.

Previous studies have shown that waterborne diseases such as typhoid fever are prevalent in many developing countries, particularly those with poor water management systems and inadequate sanitation facilities. For example, a study conducted in a rural area of Bangladesh [20] found that the incidence of typhoid fever was significantly higher in households with contaminated drinking water sources compared to those with safe drinking water sources. Other studies [5,10,11], however, have demonstrated an association between raw tap water consumption and typhoid fever, suggesting the harmfulness of public water. Consumption of raw fruit and vegetables from family gardens was found to be a risk factor for typhoid fever. Most probably, these vegetables were contaminated by wastewater with which 11% of family gardens were irrigated, or through human handling during the harvesting and consumption process. Indeed, typhoid fever was classified as a disease associated with the use of domestic wastewater [21]. The third environmental factor independently associated with typhoid fever was the uncontrolled dumping of household waste. These findings are consistent with several environmental studies [22,23] that showed that inefficient household waste management was associated with a high risk of developing the disease. In our study, we did not study the concept of washing hands because we started health and hygiene education in the community at the same time, and all households had improved their hygiene practices at the time of the study. Moreover, washing hands has been amply demonstrated in the literature [14,15,16,24] as a protective factor.

The results of the environmental and bacteriological investigations supported the hypothesis of a waterborne epidemic. Contaminated softened water proved to be the most likely vehicle for transmission of infection at the beginning of the outbreak, and the asymptomatic carrier detected among the sellers of softened water was probably the source of contamination. However, molecular typing of salmonella strains isolated from patients, asymptomatic carriers, and in water has not been done. This typing would have provided important evidence, especially in the context of an epidemic [25], to suggest that the agent found among patients is not differentiable, using the most discriminate molecular techniques, from the agent found in the softened water and among the asymptomatic carrier water manipulators.

The present study is considered to be among the first documented investigations of the outbreak of typhoid fever in Tunisia based on a multidisciplinary methodological approach; epidemiological, environmental, and microbiological factors with a descriptive and analytical dimension were analyzed to test hypotheses generated in the descriptive part. To assess the association between multiple exposures and typhoid fever we designed a retrospective cohort study that allowed us the direct calculation of attack rates and the associated measure corresponding to each factor. However, the logistic regression model used to measure the strength of the association between risk factors and disease occurrence does not provide a direct measurement of relative risk but rather the odds ratio. The odds ratio provides a reasonable approximation of the risk ratio when the health outcome is uncommon. In fact, typhoid fever is relatively rare, and the odds ratio can be used in a cohort study. The main limitation of our study was related to the lack of laboratory capacity at the regional level in terms of typing and molecular microbiology; a comparison of human strains with each other and with those isolated in asymptomatic carriers and in environmental samples was not performed.

The primary reason for any outbreak investigation is to implement the most appropriate control measures in order to prevent future outbreaks, and the implementation of specific recommendations must therefore be guided by the results of the epidemiologic investigation.

In our context, the initial measures were first focused on the health and hygiene education of the local population to break the chain of transmission of the disease by washing hands thoroughly and frequently using soap, in particular after having been to the toilet or before eating. Other, more specific measures have also been taken, such as the immediate closure of clandestine sale units of softened water in the region, the reinforcement of food and water safety inspections in restaurants, and of street food vendors’ activities. Finally, coordination with local and regional authorities was recommended to improve living conditions, to ensure a safe water supply, adequate sanitation, and proper disposal of household waste.

In addition, this epidemic episode was also an opportunity to improve our routine surveillance system by developing a protocol for typhoid fever outbreak investigation and creating regional health monitoring units for the early detection of any health event and its reporting at the national level, in order to optimize epidemic response and control.

## 5. Conclusions

This investigation revealed an extended outbreak of typhoid fever in a small region in the south of Tunisia and linked it to the consumption of softened water from clandestine sale units, fruit and vegetables from family gardens, uncontrolled dumping of household waste, and poor socio-economic conditions. Control and prevention measures have been implemented, namely health and hygiene education, and abstention from obtaining drinking water from clandestine sale units of softened water and from street vendors. The study outcome also suggested the immediate closure of these sale units in the Ghannouche region based on existing legislation, as well as the reinforcement of food outlet and drinking water system inspections.

Possible recommendations to prevent future outbreaks could involve improving the overall sanitation and hygiene situation in the identified hotspots of TF transmission by hygiene promotion. Moreover, an improved drinking water supply network, optimal chlorination of tap water, and regular monitoring of chlorination levels should be carried out to improve the situation. However, a further environmental study of water quality needs to be elaborated to analyze the reasons why households avoided tap water and consumed water from other sources. Additionally, it is necessary to complete the investigations with molecular typing to characterize the salmonella strain responsible for this epidemic in order to formulate the appropriate therapy.

## Figures and Tables

**Table 1:** Specific attack rate by sub groups, Ghannouche, July–September 2016.

Sub Groups	Exposed Population	Number of TF Cases (Proportional Distribution of Cases (%))	Attack Rate (%) (CI_95%_)
Age (years)	*p* < 10^−3^		
<10	102	39 (38.24)	38.23 (28.9–47.7)
10–20	137	29 (28.43)	21.16 (14.5–27.8)
20–30	117	14 (13.73)	11.96 (6.5–18.1)
30–40	93	9 (8.82)	9.67 (4.3–16.3)
40–50	88	7 (6.86)	7.95 (3.1–14.3)
50–60	58	3 (2.94)	5.17 (0.0–11.3)
≥60	33	1 (0.98)	3.03 (0.0–10.7)
Gender	NS		
Male	317	58 (56.86)	18.29 (14.0–22.7)
Female	311	44 (43.14)	14.14 (10.6–18.2)
Level of education	*p* < 10^−3^		
Illiterate	42	4 (3.92)	9.52 (2.2–20.0)
out-of-school	64	35 (34.31)	54.68 (37.1–61.0)
Primary	209	41 (40.20)	19.61 (14.8–26.0)
Secondary	244	17 (16.67)	6.96 (4.0–10.3)
University	69	5 (4.90)	7.24 (1.6–14.1)
Housing type	*p* < 10^−3^		
Arab or rudimentary house	234	80 (78.43)	34.18 (26.3–37.8)
Villa or apartment	394	22 (21.57)	5.58 (4.6–10.6)
Total	628	102 (100)	16.24 (13.2–19.3)

**Table 2:** Clinical signs observed among typhoid fever cases, Ghannouche, July–September 2016.

Clinical Signs	Number N = 102	Percentage (%)
Fever	97	95.1
Diarrhea	58	56.9
Abdominal pain	48	47.1
Vomiting	47	46.1
Asthenia	43	42.2
Constipation	10	9.8
Tuphos or neurological signs	4	3.9
Skin rash	3	2.9
Lenticular rosy spots	2	2.0

**Table 3:** Univariate analysis of the main factors associated with contracting typhoid fever in Ghannouche.

Socio-Demographic Factors Associated with Typhoid Fever	Crude OR	CI _95%_	*p*
Age groups (years)			
<10	19.81	(2.60–150.83)	0.004
(10–20)	8.59	(1.12–65.56)	0.038
(20–30)	4.35	(0.55–34.37)	0.163
(30–40)	3.42	(0.41–28.16)	0.251
(40–50)	2.76	(0.32–23.38)	0.35
(50–60)	1.74	(0.17–17.49)	0.636
≥60 (ref)	1		
Level of education			
Illiterate	1.34	(0.34–5.32)	0.671
Out-of-school	12.10	(4.36–33.60)	<10^−3^
Primary	3.28	(1.24–8.67)	0.017
Secondary	0.95	(0.34–2.70)	0.936
University (ref)	1		
Housing type			
Arab or rudimentary house	7.55	(4.55–12.52)	<10^−3^
Villa or apartment (ref)	1		
Environmental factors associated with typhoid fever	Crude OR	CI _95%_	*p*
Type of drinking water			
Softened water	3.80	(1.80–8.05)	0.001
Private tank	1.20	(0.24–6.05)	0.818
Mineral water	1.24	(0.31–4.93)	0.757
Other (wells. cisterns. natural source)	1.03	(0.120–8.90)	0.974
Public tap water (National Water Supply and Distribution Company) (ref)	1		
Exposure to softened water/type of use			
For drinking			
Yes	3.50	(1.93–6.31)	<10^−3^
No (ref)	1		
Washing of raw vegetables			
Yes	2.24	(1.22–4.12)	0.009
No (ref)	1		
Cooking			
Yes	2.37	(1.54−3.65)	<10^−3^
No (ref)	1		
Conditions of water storage			
Covered			
No	79.05	(30.14–207.26)	<10^−3^
Yes (ref)	1		
Disinfected			
Yes	5.25	(2.60–10.66)	<10^−3^
No (ref)	1		
Exposed to sunlight			
Yes	7.62	(4.76–12.20)	<10^−3^
No (ref)			
Controlled bacteriologically			
No	2.92	(1.15–7.45)	0.0024
Yes (ref)	1		
Sanitation & household waste management			
Connection to the public sanitation system			
No	2.14	(1.38–3.32)	0.001
Yes (ref)	1		
Using cesspools			
Yes	2.46	(1.58–3.82)	<10^−3^
No (ref)	1		
Household waste management			
Uncontrolled waste disposal	5.30	(3.27–8.60)	<10^−3^
By the municipal departments (ref)	1		
Fruit & vegetable supplies			
Family garden	8.11	(5.10–12.91)	0.001
local market	1		

**Table 4:** Independent factors associated with developing typhoid fever in Ghannouche.

Factors	AOR	95% CI	*p*
Age	0.95	0.93–0.97	<10^−3^
Education level			0.001
Illiterate	2.55	0.49–13.10	0.263
Out-of-school	4.76	1.34–16.81	0.015
Primary	2.16	0.72–6.48	0.168
Secondary	0.75	0.24–2.34	0.622
Living in arab or rudimentary house	4.93	2.61–8.27	<10^−3^
Drinking softened water	2.64	1.16–4.82	0.018
Fruit and vegetables from family garden	6.13	3.66–11.06	<10^−3^
Uncontrolled dumping of household waste	3.52	2.03–6.94	<10^−3^

## Data Availability

Data are available at the National Observatory of New and Emerging Diseases server [26].
